# Lightweight Vapor-Permeable Plasters for Building Repair Detailed Experimental Analysis of the Functional Properties

**DOI:** 10.3390/ma14102613

**Published:** 2021-05-17

**Authors:** Martina Záleská, Milena Pavlíková, Adam Pivák, Anna-Marie Lauermannová, Ondřej Jankovský, Zbyšek Pavlík

**Affiliations:** 1 Department of Materials Engineering and Chemistry, Faculty of Civil Engineering, Czech Technical University in Prague, Thákurova 7, 166 29 Prague 6, Czech Republic; martina.zaleska@fsv.cvut.cz (M.Z.); milena.pavlikova@fsv.cvut.cz (M.P.); adam.pivak@fsv.cvut.cz (A.P.); 2 Department of Inorganic Chemistry, Faculty of Chemical Technology, University of Chemistry and Technology, Technická 5, 166 28 Prague 6, Czech Republic; anna-marie.lauermannova@vscht.cz (A.-M.L.); ondrej.jankovsky@vscht.cz (O.J.)

**Keywords:** lightweight plasters, perlite, vapor permeability, salt crystallization resistance, water and salt transport properties

## Abstract

Three types of lightweight plasters for building repair were prepared and tested. The composition of plasters was designed in respect to their compatibility with materials used in the past in historical masonry. For the hardened plasters, detailed testing of microstructural and macrostructural parameters was realized together with the broad experimental campaign focused on the assessment of mechanical, hygric, and thermal properties. As the researched plasters should find use in salt-laden masonry, specific attention was paid to the testing of their durability against salt crystallization. The mechanical resistance, porosity, water vapor transmission properties, and water transport parameters of all the researched plasters safely met criteria of WTA directive 2-9-04/D and standard EN 998-1 imposed on repair mortars. Moreover, the tested materials were ranked as lightweight plasters and due to their low thermal conductivity they can be used for the improvement of thermal performance of repaired masonry. The salt crystallization test caused little or no damage of the plasters, which was due to their high porosity that provided free space for salt crystallization. The developed plasters can be recommended for application in repair of damp and salt masonry and due to their compatible composition also in historical, culture heritage buildings. The added value of plasters is also their good thermal insulation performance.

## 1. Introduction

Many different requirements are placed on the properties of both interior and exterior plasters. Nevertheless, their thermal insulation function has gained importance in recently. As the consumption of energy used for the temperature control in both residential and commercial buildings through heating and air conditioning is still increasing [1], the energy performance of buildings represents the driving force for the improvement and the design of the advanced thermal insulation materials. Worldwide, the buildings and construction sector were responsible for 36% of the total energy use and 39% of energy and process-related CO_2_ emissions in 2018 [2]. In the European Union (EU), the heating and cooling of buildings represents around half of the EU’s final energy consumption and is the biggest energy end-use sector, ahead of transport and electricity. Moreover, 85% of the energy used for heating and cooling is produced from natural gas, coal, and oil products and only 15% is generated from renewable energy sources [3]. The key targets for 2030 adopted by the European Commission under the 2030 Climate and Energy Framework included at least 40% cuts in greenhouse gas emissions (from 1990 levels) and at least 32.5% improvement in energy efficiency [4].

The most common way how to reduce the energy demands for heating of older buildings is application of the External Thermal Insulation Composite Systems (ETICS). This solution enables to effectively insulate the whole envelope of the building and reduce thermal bridges that would negatively affect the overall building’s hygrothermal performance [5,6,7]. However, in the case of older and historical buildings, application of ETICS is often forbidden especially due to the requirements of culture heritage authorities that insist on the preservation of the original and decorative appearance of the architectural style of facades and their elements, such as balusters, on brackets balconies, ornamental brackets, pillars and pilasters, gables, etc. Therefore, the compromise solution in the improvement of the hygrothermal performance of such buildings is to enhance their envelopes with appropriate thermal insulation plasters possessing other advanced functional parameters [8]. Many investigations were carried out focused on the integration of different kinds of insulating materials into the composition of plasters in order to achieve their low thermal conductivity and thus thermal insulation efficiency [9,10,11,12,13,14,15,16,17,18,19,20,21]. Generally, the authors reported that the addition of lightweight aggregates or fibers into plaster composition greatly improves thermal conductivity, increases porosity, and decreases the mechanical parameters of the hardened plasters. Improvements in sorption properties and water vapor permeability were also reported.

The main aim of this paper is the comprehensive analysis of three types of novel lightweight thermal insulation plasters with enhanced composition containing expanded perlite and designed for interiors, including areas with increased humidity, such as kitchens and bathrooms, or exteriors. The plasters should find use also in repair of historical masonry, where they should act as durable materials for the moderation of the indoor climate of cultural heritage buildings. During restoration works, the compatibility between the new repair mortars and the original components is essential for an adequate intervention on the monument. As pure Portland cement mortars are incompatible with most of the traditional materials inbuilt in historical structures, lime–, natural hydraulic lime–, lime–pozzolan–, gypsum–, and cement–lime-based mortars are considered as materials applicable for the repair of historical masonry and structures. In this sense, the two cement–lime plasters and one lime–gypsum plaster with expanded perlite were characterized in terms of their structural, mechanical, thermal, and hygric parameters. As the durability of plasters is a very important parameter especially in their use in restoration of salt-laden masonry, the tests of salt transport properties were conducted together with the analysis of salt crystallization resistance. To the best of the authors’ knowledge, such comprehensive analysis of plasters intended to be used for complex solution of thermal insulation and repair problems was not presented yet and can be considered as a further step for the improvement of eco-efficiency of both contemporary and older building stock. Moreover, the increasing interest in the knowledge of the properties of mortars for restoration purposes justifies the research carried out.

## 2. Materials and Methods

### 2.1. Composition of Researched Plasters

Plaster mixtures were prepared from hydrated lime CL 90-S (Čertovy Schody Inc., Tmaň, Czech Republic, member of the Lhoist group), Portland cement CEM I 42.5 R (Českomoravský cement Inc., Mokrá-Horákov, Czech Republic, member of the HeidelbergCement Group), and gypsum binder (Gypstrend Ltd., Kobeřice, Czech Republic). As aggregates, washed quartz sand of 0/1 or 0/2 mm fraction delivered from Filtrační písky, Ltd., Chlum u Doks, Czech Republic (loose bulk density 1668 kg·m^−3^) and expanded perlite EP 150 PB (Perlit Praha Ltd., Praha, Czech Republic) of fraction 0/1 or 0/2 mm and having loose bulk density of 178 kg·m^−3^, were used. Due to the short setting times of gypsum, citric acid monohydrate (Inchema Ltd., Horní Počernice, Czech Republic) was added as a setting retarder.

The chemical composition of the employed materials was obtained from X-Ray Fluorescence (XRF) analysis which was conducted by an ARL QUANT’X EDXRF Spectrometer (Thermo Scientific, Madison, WI, USA), equipped with a Rh X-Ray tube and Si(Li) detector crystal. The data were collected and evaluated using the UniQuant ED 6.32 software (Thermo Scientific, West Palm Beach, FL, USA).

The CLM1 plaster was prepared with a binder-to-aggregate volume ratio of 1:1.15. This volume ratio corresponds with the 1:4 weight ratio commonly used in preparation of lime and cement–lime renders in research and practice [22,23]. This binder to aggregate ratio was also well documented in historical masonry [24,25,26]. Similar dosage of the blended binders and particular aggregates was then followed in CLM2 and LGM plasters. In the preparation of samples, the use of a constant binder/aggregate volume ratio guarantees an equal proportion of binder and aggregate in the individual mixtures for materials with different loose bulk densities. This is especially important when using lightweight aggregate, such as perlite, where the amount of aggregate would increase disproportionately at a constant weight ratio.

The volume of water was adjusted to maintain the similar and normal consistency of all plasters; flow 160 ± 5 mm. The flow was verified in accordance with EN 1015-3 [27]. Based on the research on gypsum-based mortars reported in [28], the amount of citric acid was of 0.03 wt. % of the dry compounds. The composition of the investigated plasters is given in Table 1. The casted specimens were demolded after 48 h and stored at temperature 23 ± 2 °C and relative humidity 50 ± 5% until their testing. The casted samples were 40 mm × 40 mm × 160 mm prisms, 100 mm cubes, and circular plates having a radius of 120 mm and thickness of 30 mm.

For the dry plaster mixtures, the particle size was measured using a standard sieve analysis. The sieves with apertures of 0.063, 0.09, 0.125, 0.25, 0.5, 1.0, and 2.0 mm were used.

The 28-days hardened samples were tested. For each batch of plaster, a minimum of 5 samples were examined.

### 2.2. Assessment of Structural and Microstructural Parameters

Among the macrostructural parameters, bulk density, specific density, and total open porosity were measured. The dry bulk density *ρ*_b_ (kg·m^−3^) of dry renders was determined according to the European standard EN 1015-10 [29]. The samples were dried in the vacuum condition at 60 °C. The specific density *ρ*_s_ (kg·m^−3^) was assessed using a helium pycnometer Pycnomatic ATC (Porotec, Hofheim, Germany). Based on the knowledge of dry bulk density and specific density values, the total open porosity *ψ* (-) was calculated [30]. The expanded combined uncertainties of the bulk density, specific density, and porosity determination were 1.4%, 1.2%, and 2.0%. The microstructure of the investigated plasters was analyzed by mercury intrusion porosimetry (MIP), which was conducted by the use of porosimeters of Pascal series, Pascal 140 and Pascal 440 (Thermo Fisher Scientific, Waltham, MA, USA). The measured parameters were average pore diameter, total pore volume, and incremental and cumulative pore volume distributions. The typical dry sample mass in MIP test was approx. 1.0 g.

### 2.3. Analysis of Mechanical Parameters

Flexural strength, compressive strength, and dynamic modulus of elasticity were the researched mechanical parameters. The strength tests were realized according to the standard EN 1015-11 [31]. In the three-point bending test, the flexural strength *f*_f_ (Mpa) was measured on the standard 40 mm × 40 mm × 160 mm prisms. The load speed was 50 N·s^−1^. The compressive strength *f*_c_ (Mpa) was measured on the fragments from the flexural strength test. The uniaxial compression force (100 N·s^−1^) was applied on the 40 mm × 40 mm cross section of the specimens. For the dynamic modulus of elasticity *E*_d_ (Gpa) measurement, a Vikasonic apparatus (Schleinbinger Geräte, Buchbach, Germany) was utilized. The mechanical parameters were measured on 5 samples of the particular tested plaster. The expanded combined uncertainty of the mechanical parameters assessment was 1.4%, 1.4%, and 2.3% for *f*_f_, *f*_c_, and *E*_d_, respectively.

### 2.4. Determination of Thermal Parameters

Heat transport and storage parameters of dry hardened samples were determined by a thermal constants analyzer ISOMET 2114 (Applied Precision, Bratislava, Slovakia) operating on a transient impulse technique principle [32]. For the measurement, circular surface probe IPS 1105 was used. The measurement range of the applied probe was 0.04–0.3 W·m^−1^·K^−1^ for the thermal conductivity λ (W·m^−1^·K^−1^) and 4.0 × 10^4^–1.5 ×10^6^ J·m^−3^·K^−1^ for the volume heat capacity *c*_v_ (J·m^−3^·K^−1^).

### 2.5. Hygric Parameters

The water vapor transmission rate in the examined plasters was quantified using a cup method in both wet-cup and dry-cup arrangements [33]. The tests were conducted according to EN ISO 12572 [34]. For the cup measurements, the circular plate samples having diameter of 120 mm and thickness of 30 mm were conditioned at constant temperature *T* = (23 ± 0.5) °C and relative humidity *RH* = (50 ± 5)% until they reached constant mass. The samples were placed in steel cups and then sealed by technical plasticine to ensure 1-D water vapor transport through their exactly known cross-sectional area. In the wet-cup test, the cup contained saturated solution of KNO_3_ that generated below the specimen placed in cup RH of (93 ± 5)%, while in the dry-cup measurement the cup contained silica gel that maintained RH of approx. 2%. The upper side of the specimen was exposed to RH of (50 ± 5)% which was maintained by an automatically controlled climate chamber. From the measured steady-state specimen mass gain (dry cup) or loss (wet cup), water vapor permeability *δ* (s) of a particular plaster was obtained. The water vapor permeability of air *δ*_a_ (s) was determined based on the temperature and atmospheric measurements using Schirmer’s equation [35]. The water vapor resistance factor *μ* (-) was calculated as *δ*_a_/*δ* ratio. The duration of the cup test was approximately 8 days depending on the material permeability for water vapor. The expanded combined uncertainty of the water vapor diffusion test was for the water vapor permeability and the water vapor resistance factor 2.8%.

The plasters’ water vapor adsorption capacity was characterized by the measurement of sorption and desorption isotherms. The samples were placed in a set of desiccators that contained saturated solutions that enabled to maintain selected RH. To acquire required relative humidities that covered the whole hygroscopic moisture range, following salts were used: LiCl, K_2_CO_3_, NaCl, KCl, and K_2_SO_4_. At the maintained constant temperature *T* = (23 ± 0.5) °C, these salts provided RH of 11%, 43%, 75%, 85%, and 98%, respectively. In every desiccator, 3 dry specimens of each studied plaster were placed on the plastic grid above the saturated salt solution. The 40 mm × 40 mm × 10 mm samples were cut from the standard prisms. The mass of the specimens was monitored until they mass difference was <0.1%. Then, the moisture content by mass was calculated, statistically evaluated, related to the corresponding RH, and one point of sorption/desorption isotherm was plotted. In this way, the adsorption and desorption isotherms were constructed based on the static gravimetric method [36]. The hysteresis in desorption process [37] was also monitored.

The water absorption coefficient *A*_w_ (kg·m^−2^·s^−1/2^) and 24-h water absorption *W*_a_ (kg·m^−2^) was assessed in accordance with the EN 1015-18 [38]. The 40 mm cubes were on lateral sides insulated by epoxy resin and their bottom side submerged 5 mm in water. The 24-h water absorption and water absorption coefficient were measured not only for penetration of water, but also for 1% NaCl and 1% Na_2_SO_4_ water solutions. The expanded combined uncertainty of both water absorption tests was 1.2%.

### 2.6. Salt Crystallization Resistance

As to date no commonly accepted methodology for the accelerated salt aging test of porous building materials is available, the testing procedure for the assessment of salt crystallization resistance was originally designed based on the standard EN 12370 [39]. In order to reflect the real situation in practice, two salts were chosen for the test procedure, namely sodium sulfate (anhydrous) Na_2_SO_4_ and sodium chloride NaCl, and moreover, the concentration of both salts was lower than prescribed in the standard EN 12370 [39], which overestimates salt concentration in the crystallization experiment. According to the recommendations of Lubelli et al. [40] and Granneman et al. [41], the amount of each salt used was chosen to be 2% (weight salt/weight dry specimen). Oven dried specimens having dimensions of 40 mm × 40 mm × 40 mm were subjected to 10 crystallization cycles, each cycle consisted of samples immersion in salt solution for 2 h followed by drying in an oven at 70 °C for at least 16 h. After drying, specimens were removed from the oven and left to cool for 2 h. Each sample was placed in its own container, which was during the immersion and cooling phase covered with the cap to prevent evaporation. For the evaluation of the salt crystallization effect, visual observation, light microscopy (LM) analysis, compressive strength tests, and ultrasonic measurement were conducted. The Light Microscopy (LM) was performed by Navitar (Rochester, NY, USA) macro-optics with optical zoom up to 110X and recorded with digital camera Sony 2/3″, with a resolution of 5 Mpix. The compressive strength and the dynamic modulus of elasticity tests were conducted in a similar way as described above. Moreover, loss or gain of specimen mass after crystallization cycles was recorded, similarly as prescribed in EN 12370 [39]. Based on the performed experiments, the compressive strength and the dynamic modulus of elasticity ratios of samples that underwent salt crystallization and reference samples were assessed.

Table 2 summarizes a nomenclature of symbols used.

## 3. Results and Discussion

The chemical composition of the employed materials obtained by XRF is introduced in Table 3.

**Figure 1:**
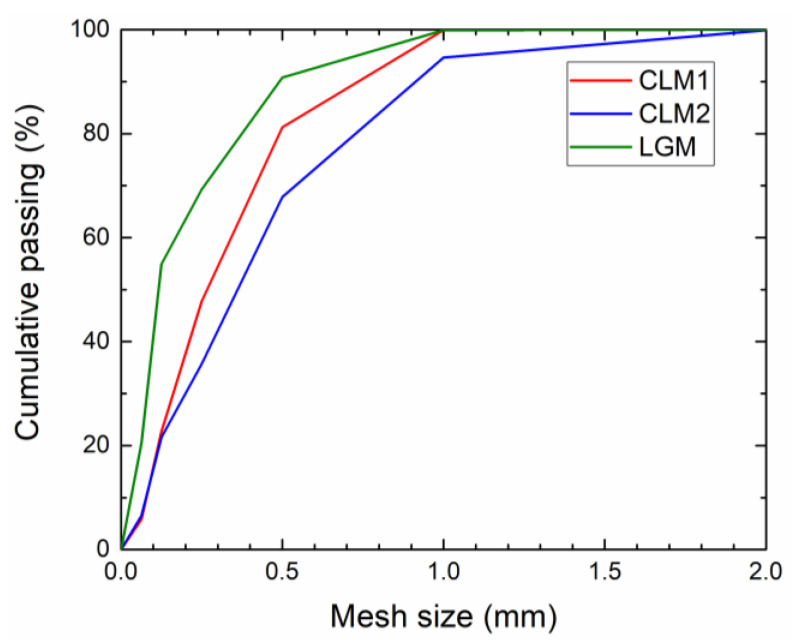
Particle size distribution of the analyzed plasters.

The particle size distribution curves of the prepared dry plasters mixtures are plotted in Figure 1. The recorded particle size distribution corresponds to the particle size of raw materials contained in plasters’ composition and their dosage.

The results of the conducted tests and analyses were evaluated using the specifications for masonry and repair mortars summarized in EN 998-1 [42] and the WTA directive 2-9-04/D [43].

The macrostructural parameters of the investigated plasters are summarized in Table 3. The agreement between the bulk density and total open porosity values is quite obvious. The WTA directive 2-9-04/D [43] prescribes the bulk density < 1400 (kg·m^−3^) for the repair plaster. Similarly, the porosity must be >40%. Both these conditions were safely encountered for all tested materials. In Table 4, the total open porosity measured by mercury intrusion *ψ_Hg_* (-) is also presented. Taking into consideration the principles of the applied total porosity assessment methods, low weight of samples for MIP tests, and inhomogeneity of the tested plasters, the difference in the porosity values can be considered as insignificant.

**Figure 2:**
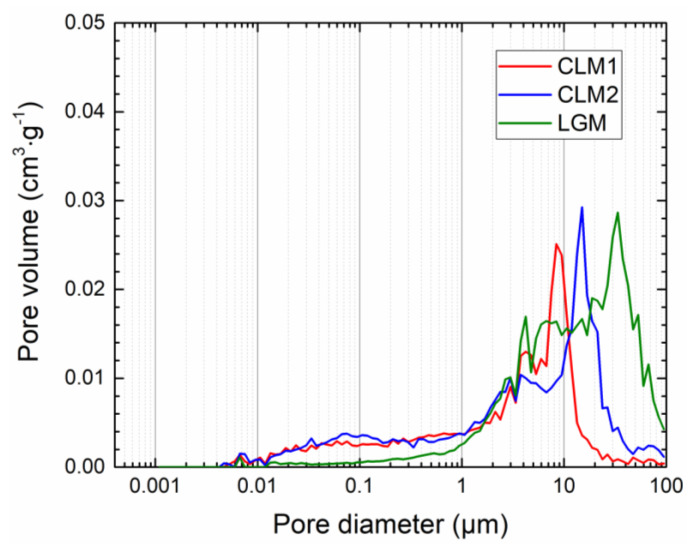
Incremental pore volume distribution of the researched plasters.

The pore size distribution curves obtained by mercury intrusion porosimetry are given in Figure 2 and Figure S1 (Supplementary Materials). Apparently, the microstructural data corresponds with the total porosity data presented in Table 4. The pore size distribution parameters acquired by MIP are presented in Table S1 (Supplementary Materials). They clearly characterize highly open porous structure of the examined materials, which is positive with respect to the requirements imposed on the repair plasters. In the whole studied pore diameter range, the volume of pores was the lowest for plaster CLM1. Contrary to that the relative volume of pores in the recorded pore radii was the highest for LGM. The relative volumes of pores of plaster CLM2 were in the middle.

The mechanical resistance of the investigated plasters is apparent from Table 5. These are the results of three combined effects, the nature of the used binder, use of lightweight admixture (perlite), and porosity. The highest flexural strength and compressive strength exhibited lime–gypsum plaster LGM. On the other hand, the dynamic modulus of elasticity of this material was in the middle. Similar performance of lime–gypsum plasters was reported, e.g., in [44,45]. From the practical point of view, all plasters can be classified in category CS II [42] which well satisfies the condition of the WTA directive 2-9-04/D [43]. According to this directive, the compressive strength of repair plasters must be in the 1.5–5.0 MPa. This criterion safely met all prepared plastering mortars.

Heat transport and storage in the investigated plasters were characterized by the thermal conductivity *λ* (W·m^−1^·K^−1^), thermal diffusivity *a* (m^2^·s^−1^), and volumetric heat capacity *c*_v_ (J·m^−3^·K^−1^). These parameters are summarized in Table 6. As there is not any strict requirement on the thermal characteristics of plaster intended for repair applications, the thermal insulation potential was evaluated in respect to EN 998-1 [42]. According to this standard, all tested plasters can be ranked as lightweight plasters for interior and in the case of CLM materials also for exterior use. Moreover, lime–gypsum plaster satisfied the criteria imposed on thermal insulation plaster of T2 type. The thermal conductivities of both cement–lime plasters were slightly higher than required limit for T2 (*λ* < 0.2), but they were still acceptable for the improvement of thermal performance of repaired masonry.

The water vapor transmission properties are important parameters of plasters for repair applications. The results of the cup test that was conducted in both dry cup and wet cup arrangements are presented in Table 7. All materials had the water vapor resistance factor <12.0, which is strictly limited by the WTA directive 2-9-04/D [43]. Such plasters enable water vapor release from the interior and possible drying of the structures suffering from the excessive moisture presence. It must be noted, the water vapor resistance factor criterion of the WTA directive is higher than that prescribed for repair mortars in the EN 998-1 [42] which requires *µ* < 15.0. The water vapor permeability was higher for wet cup arrangement of the test than that of assessed in the dry cup analysis. Similar material performance in the different conditions of the cup experiment was observed, e.g., in [46,47,48]. The acceleration of water vapor transmission in the wet cup test can be attributed to the reduced surface binding forces between water vapor molecules and pores due to the filling by water molecules within samples conditioning for the test [49].

Basically, the water vapor permeability of materials is considered to be dependent on its macrostructural and microstructural parameters and binder nature [50,51]. In our case, not only the total pore volume, but also pore size distribution, their shape, and tortuosity played a role in the water vapor transmission process [52]. Quantitatively, both the examined vapor transport parameters were similar to those published in [53,54,55,56,57,58].

**Figure 3:**
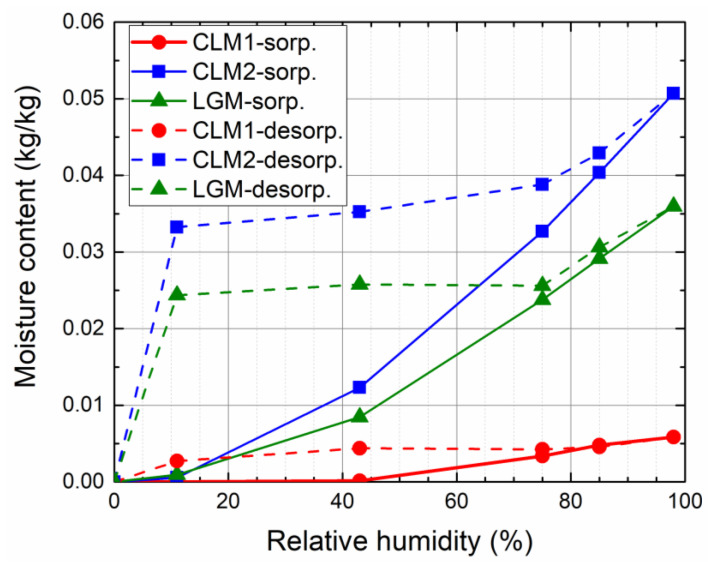
Sorption and desorption isotherms of the researched plasters.

The sorption and desorption isotherms are plotted in Figure 3. Both the sorption and desorption curves obtained for the tested materials are quite different. Based on IUPAC isotherm classification [59], which provides fundamental guidance how to interpret sorption isotherms for the purpose of structural characterization, the measured sorption/desorption data corresponds to the Type IVa isotherm, typical for mesoporous materials. In the case of a Type IVa isotherm, capillary condensation is accompanied by hysteresis. This occurs, when the pore width exceeds a certain critical width, which is dependent on the adsorption system and temperature.

As expected, the lowest water vapor absorption capacity exhibited plaster CLM1; up to 43% of relative humidity (RH), the gravimetric water content was in the range of detection error. For the higher relative humidity, the moisture content increases slightly versus RH to reach about 0.6%. Plasters CLM2 and LGM were more sensitive to the RH changes of the environment. Since RH > 40%, the capillary condensation arose [60], the micropores and mesopores were filled by water molecules [61], and the water content has strongly increased to reach about 5.1% and 3.6% at 98% RH for plasters CLM2 and LGM, respectively.

The hysteresis was well visible for all researched plasters. In case of CLM1, it reached about 0.4% and it was almost constant in the RH range 11–80%. The hysteresis of CLM2 was in the range 4.3–3.3%, and the desorption curve exhibited linearly decreasing character in the RH range 11–75%. The hysteresis of LGM was about 2.5% and was almost unchanged in the RH range 11–75%. The observed hysteresis in desorption process is usually assigned to the capillary condensation hysteresis [62], the contact angle hysteresis [63], the ink-bottle effect [64], or chemical interaction of material with water molecules [65]. A part of the residual moisture may also be due to the partial lime carbonation that could start at high relative humidity. Similar residual mass of lime-based plasters observed, e.g., Mazhoud et al. [58].

The 24 h water absorption *W*_w_ and water absorption coefficient *A*_w_ assessed in accordance with EN 1015-18 [38] are presented in Table 8. All studied plasters exhibited high water absorption capability and safely met criteria of water absorption rate prescribed in EN 998-1 [42] and WTA directive 2-9-04/D [43]. According to EN 998-1 [42], the repair mortar must have *W*_a_ ≥ 0.3 kg·m^−3^, which was well fulfilled. Quantitatively, the water ingress into the studied plasters corresponded with the pore size distribution and microstructural parameters, which were the determining factors affecting the overall water imbibition. Namely the volume of capillary pores (0.01–10 µm) in the particular plasters affected the water absorption rate and the total water ingress. Therefore, not the total open porosity, but the share of the volume of capillary pores on overall porosity was the dominant parameter for moisture transport. Similarly, the transport of the tested salt solutions was also governed by the porous structure parameters.

The differences between the observed hygric parameters assessed for penetration of tap water and 1% NaCl and Na_2_SO_4_ solutions were small, but some deceleration in the transport of salt solutions in comparison with the transport of pure water can be distinguished.

In Table 9, the mass change of samples subjected to the salt crystallization tests is given. The ratios of the compressive strength and dynamic modulus of samples exposed to 10 wetting drying cycles (water, NaCl, Na_2_SO_4_) and that of the reference samples are also introduced. The examined plasters showed excellent resistance against crystallization of NaCl and Na_2_SO_4_ solutions. Both CLM plasters exhibited even improved compressive strength after they underwent crystallization tests and wetting/drying cycles. This was assigned to the continuous hydration of cement/lime binder whose positive contribution to the total mechanical strength prevailed against other effects. The resistance against salt crystallization was caused by the high open porosity of prepared plasters that enabled crystallization of salts from applied solutions in the free porous space without causing damage. Quantitatively bigger problem for the durability of the tested materials appeared for the action of NaCl solution that caused the biggest mass loss and drop in dynamic modulus of elasticity ratio for all the plasters. However, the damage parameters were in this case also small. In summary, considering the results of salt crystallization tests and measured plasters’ residual parameters, the prepared plasters can be recommended as plastering materials for salt laden masonry.

**Figure 4:**
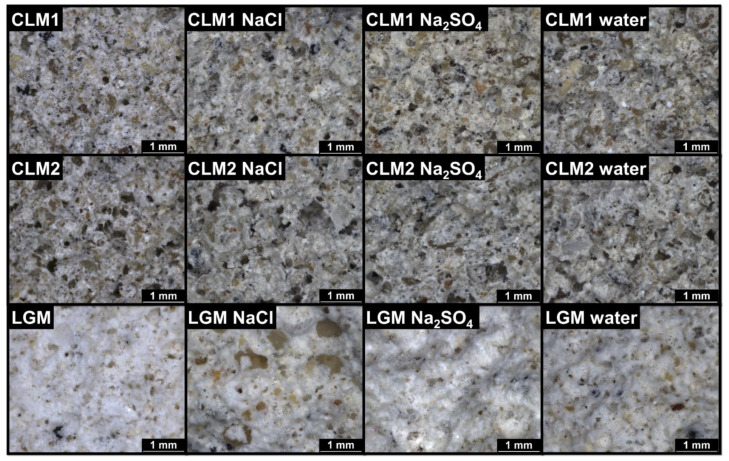
Structure of the hardened plasters analyzed by light microscopy.

The results of light microscopy imaging are introduced in Figure 4. No cracks or any surface damage was observed on the plasters’ fracture surface, which proves the high salt crystallization resistance of the analyzed materials. Photographical observation has not detected any damage of samples subjected to the salt crystallization test as apparent from Figure S2 (Supplementary Materials).

## 4. Conclusions

Lightweight vapor permeable plasters for building repair were designed and tested within the broad experimental campaign. The conducted complex research involved chemical analysis of the base materials, standard sieve analysis of plasters dry mixtures, measurement of macrostructural, microstructural and mechanical properties, and assessment of hygrothermal parameters of the hardened plasters. Specific attention was paid to the analysis of the salt crystallization resistance and determination of the residual strength and stiffness after the wetting/drying cycles in the condition of water, NaCl and Na_2_SO_4_ solutions. The following main results were obtained and highlighted:

(i) The bulk density and porosity of the developed plasters met requirements prescribed for repair mortars;
(ii) Based on the compressive strength values, all plasters were classified in category CS II, which is positive with respect to the encountered requirements imposed on the repair plastering materials;
(iii) The tested plasters can be ranked as lightweight plasters for interior or exterior application (CLM materials). Lime–gypsum plaster was classified as the thermal insulation plaster of T2 type. Although the thermal conductivities of cement–lime plasters slightly exceed the limit for T2, they are still applicable for the improvement of the thermal insulation of repaired masonry;
(iv) The plasters were found highly permeable for water vapor, which enables the drying of the treated substrates suffering from the excessive moisture action;
(v) All studied plasters exhibited high water absorption capability and safely met criteria of the water absorption rate;
(vi) Considering the results of salt crystallization tests and measured plasters’ residual parameters, the prepared plasters can be recommended as plastering materials for masonry suffering from salt action.

With respect to the obtained experimental results, it was summarized that the developed materials can find use as lightweight repair plasters possessed of sufficient mechanical strength, high water vapor and water permeability, thermal insulation performance, and durability against salt crystallization and cyclic wetting/drying. They can be therefore applied on salt-laden masonry, and in case of the excessive moisture presence will ensure water evaporation and thus drying the repaired masonry.

## Figures and Tables

**Table 1:** Composition of the investigated plasters.

Plaster	Lime (g)	Cement (g)	Gypsum (g)	Sand 0/1 (g)	Sand 0/2 (g)	Perlite (g)	Citric Acid (g)	Water (g)
CLM1	50	50	-	70	-	75	-	30
CLM2	45	55	-	-	70	75	-	28
LGM	30	-	70	70	-	75	0.07	45

**Table 2:** The nomenclature of used symbols.

Parameter	Symbol	Unit
Specific density	*ρ* **_s_**	(kg·m^−3^)
Bulk density	*ρ*_b_	(kg·m^−3^)
Total open porosity	*ψ*	(%)
Hg porosity	*ψ_Hg_*	(%)
Flexural strength	*f*_f_	(MPa)
Compressive strength	*f*_c_	(MPa)
Dynamic modulus of elasticity	*E*_d_	(GPa)
Thermal conductivity	*λ*	(W·m^−1^·K^−1^)
Thermal diffusivity	*a*	(m^2^·s^−1^)
Volumetric heat capacity	*c*_v_	(J·m^−3^·K^−1^)
Water vapor permeability	*δ*	(s)
Water vapor resistance factor	*μ*	(-)
Water absorption coefficient	*A*_w_	(kg·m^−2^·s^−1/2^)
24-h water absorption	*W*_a_	(kg·m^−2^)

**Table 3:** Chemical composition of initial materials (wt.%) obtained by XRF.

Material	SiO_2_	Al_2_O_3_	Fe_2_O_3_	CaO	MgO	K_2_O	Na_2_O	P_2_O_5_	TiO_2_	SO_3_
Lime	0.23	0.13	0.16	98.74	0.43	-	-	-	-	0.13
Cement	20.35	4.91	3.24	65.29	1.47	0.91	0.14	0.08	0.45	3.13
Gypsum	6.91	2.47	1.03	42.41	0.68	0.43	-	0.05	0.27	45.60
Quartz sand	98.71	0.45	0.18	0.02	0.03	0.08	0.01	0.03	0.10	0.02
EP	67.72	18.04	1.83	4.34	0.40	2.30	4.43	0.14	0.10	0.10

**Table 4:** The macrostructural properties of the hardened plasters.

Material	*ρ*_s_ (kg·m^−3^)	*ρ*_b_ (kg·m^−3^)	*ψ* (%)	*ψ_Hg_* (%)
CLM1	2587 ± 31	1269 ± 18	50.9 ± 1.0	48.7
CLM2	2536 ± 30	1120 ± 16	55.8 ± 1.1	54.1
LGM	2389 ± 29	1046 ± 15	56.2 ± 1.1	59.7

**Table 5:** The mechanical and thermal properties of the hardened plasters.

Material	*f*_f_ (MPa)	*SD* *	*f*_c_ (MPa)	*SD* *	*E*_d_ (GPa)	*SD* *
CLM1	0.9	0.10	1.7	0.17	2.3	0.16
CLM2	1.4	0.10	2.4	0.21	3.5	0.19
LGM	1.9	0.16	3.6	0.24	2.7	0.14

* SD—standard deviation.

**Table 6:** The thermal properties of the hardened plasters.

Material	*λ* (W·m^−1^·K^−1^)	*a* (m^2^·s^−1^)	*c*_v_ (J·m^−3^·K^−1^)
CLM1	0.227	0.950 × 10^−6^	0.239 × 10^6^
CLM2	0.211	0.938 × 10^−6^	0.225 × 10^6^
LGM	0.191	0.964 × 10^−6^	0.198 × 10^6^

**Table 7:** The water vapor transport parameters of the hardened plasters.

Material	Dry Cup		Wet Cup	
	*δ* × 10^−10^ (s)	*µ* (-)	*δ* × 10^−10^ (s)	*µ* (-)
CLM1	1.73	11.4	2.63	7.5
CLM2	2.03	9.7	2.90	6.8
LGM	1.94	10.2	3.13	6.3

**Table 8:** The hygric parameters of the researched plasters.

Material	Water		NaCl		Na_2_SO_4_	
	*A*_w_ (kg·m^−2^·s^−1/2^)	*W*_a_ (kg·m^−2^)	*A*_NaCl_ (kg·m^−2^·s^−1/2^)	*W*_NaCl_ (kg·m^−2^)	*A*_Na2SO4_ (kg·m^−2^·s^−1/2^)	*W_Na2SO_*_4_ (kg·m^−2^)
CLM1	0.187	12.7	0.178	12.8	0.190	12.2
CLM2	0.149	12.4	0.114	11.7	0.113	12.1
LGM	0.121	12.0	0.110	11.5	0.109	12.0

**Table 9:** Mass loss after the salt crystallization test (wt.%), the compressive strength, and dynamic modulus of elasticity ratios of samples subjected to salt crystallization tests and reference samples.

Material	Water	NaCl	Na_2_SO_4_
CLM1	−0.80	−1.85	−0.83
CLM2	−0.41	−0.24	−0.18
LGM	−0.23	−1.02	−0.25
Compressive strength ratio (-)			
CLM1	1.01	1.08	1.19
CLM2	1.18	0.99	1.03
LGM	0.99	0.91	1.02
Dynamic modulus of elasticity ratio (-)			
CLM1	0.96	0.88	0.95
CLM2	0.98	0.89	0.99
LGM	0.85	0.90	0.93

## Data Availability

The data presented in this study are available on request from the corresponding author. The data are not publicly available due to privacy.

## Supplementary Materials

The following are available online at https://www.mdpi.com/article/10.3390/ma14102613/s1, Figure S1: Cumulative pore volume distribution of the researched plasters, Figure S2: Photos of the researched plasters before and after salt crystallization test: (**a**) Plaster CLM1; (**b**) Plaster CLM2; (**c**) Plaster LGM, Table S1: The pore size distribution parameters.

## References

[1] Abu-Jdayil B., Mourad A.-H., Hittini W., Hassan M., Hameedi S. (2019). Traditional, state-of-the-art and renewable thermal building insulation materials: An overview. Constr. Build. Mater..

[2] International Energy Agency Global Status Report for Buildings and Construction 2019. https://www.iea.org/reports/global-status-report-for-buildings-and-construction-2019.

[3] European Commission Horizon 2020 Energy Efficiency. https://ec.europa.eu/easme/en/section/horizon-2020-energy-efficiency/heating-and-cooling.

[4] Climate & Energy Framework, European Commission European Strategies and Targets. https://ec.europa.eu/clima/policies/strategies/2030_en.

[5] Shen X., Li L., Cui W., Feng Y. (2020). Thermal and moisture performance of external thermal insulation system with periodic freezing-thawing. Appl. Therm. Eng..

[6] Francke B., Zamorowska R. (2020). Resistance of External Thermal Insulation Composite Systems with Rendering (ETICS) to Hail. Materials.

[7] Michałowski B., Marcinek M., Tomaszewska J., Czernik S., Piasecki M., Geryło R., Michalak J. (2020). Influence of Rendering Type on the Environmental Characteristics of Expanded Polystyrene-Based External Thermal Insulation Composite System. Buildings.

[8] Barnat-Hunek D., Grzegorczyk-Frańczak M., Klimek B., Pavlíková M., Pavlík Z. (2021). Properties of multi-layer renders with fly ash and boiler slag admixtures for salt-laden masonry. Constr. Build. Mater..

[9] Belayachi N., Hoxha D., Slaimia M. (2016). Impact of accelerated climatic aging on the behavior of gypsum plaster-straw material for building thermal insulation. Constr. Build. Mater..

[10] Ashour T., Wieland H., Georg H., Bockisch F.-J., Wu W. (2010). The influence of natural reinforcement fibres on insulation values of earth plaster for straw bale buildings. Mater. Des..

[11] Ismail B., Belayachi N., Hoxha D. (2020). Optimizing performance of insulation materials based on wheat straw, lime and gypsum plaster composites using natural additives. Constr. Build. Mater..

[12] Maia J., Ramos N.M., De Freitas V.P., Ângela S. (2015). Laboratory Tests and Potential of Thermal Insulation Plasters. Energy Procedia.

[13] Gencel O., del Coz Diaz J.J., Sutcu M., Koksal F., Rabanal F.P.A., Martínez-Barrera G. (2016). A novel lightweight gypsum com-posite with diatomite and polypropylene fibers. Constr. Build. Mater..

[14] Dylewski R., Adamczyk J. (2014). The comparison of thermal insulation types of plaster with cement plaster. J. Clean. Prod..

[15] Corinaldesi V., Donnini J., Nardinocchi A. (2015). Lightweight plasters containing plastic waste for sustainable and energy-efficient building. Constr. Build. Mater..

[16] Petrella A., Di Mundo R., De Gisi S., Todaro F., Labianca C., Notarnicola M. (2019). Environmentally Sustainable Cement Composites Based on End-of-Life Tyre Rubber and Recycled Waste Porous Glass. Materials.

[17] Buratti C., Moretti E., Belloni E., Agosti F. (2014). Development of Innovative Aerogel Based Plasters: Preliminary Thermal and Acoustic Performance Evaluation. Sustainability.

[18] Nosrati R.H., Berardi U. (2018). Hygrothermal characteristics of aerogel-enhanced insulating materials under different humidity and temperature conditions. Energy Build..

[19] Vyšvařil M., Pavlíková M., Záleská M., Pivák A., Žižlavský T., Rovnaníková P., Bayer P., Pavlík Z. (2020). Non-hydrophobized perlite renders for repair and thermal insulation purposes: Influence of different binders on their properties and durability. Constr. Build. Mater..

[20] Fenoglio E., Fantucci S., Serra V., Carbonaro C., Pollo R. (2018). Hygrothermal and environmental performance of a perlite-based insulating plaster for the energy retrofit of buildings. Energy Build..

[21] Rashad A.M. (2016). A synopsis about perlite as building material-A best practice guide for Civil Engineer. Constr. Build. Mater..

[22] Lanas J., Alvarey-Galindo J. (2003). Masonry repair lime-based mortars: Factors affecting the mechanical behavior. Cem. Concr. Res..

[23] Horn K. (2011). Lime Rendering-Sustainable Heritage Report No. 1.

[24] Moropolou A., Cakmak A.S., Lohvyn N. (2000). Eartquake resistant construction techniques and materials on Byzantine monuments in Kiew. Soil. Dyn. Eartqu. Eng..

[25] Tenconi M., Karatasios I., Bala’awi F., Kilikoglou V. (2018). Technological and microstructural characterization of mortars and plasters from the Roman site of Qasr Azraq, in Jordan. J. Cult. Herit..

[26] Cazalla O., Rodriguez-Navarro C., Sebastian E., Cultrone G. (2000). Aging of lime putty: Effects on traditional lime mortar car-bonation. J. Am. Ceram. Soc..

[27] Methods of Test for Mortar for Masonry (1999). Part 3: Determination of Consistence of Fresh Mortar (by Flow Table).

[28] Krejsová J., Doležalová M., Pernicová R., Vimmrová A. (2018). The influence of different aggregates on the behavior and properties of gypsum mortars. Cem. Concr. Compos..

[29] (1999). Methods of Test for Mortar for Masonry—Part 10: Determination of Dry Bulk Density of Hardened Mortar.

[30] Záleská M., Pavlík Z., Čítek D., Jankovský O., Pavlíková M. (2019). Eco-friendly concrete with scrap-tyre-rubber-based aggregate-Properties and thermal stability. Constr. Build. Mater..

[31] (1999). Methods of Test for Mortar for Masonry—Part 11: Determination of Flexural and Compressive Strength of Hardened Mortar.

[32] Pavlík Z., Trník A., Keppert M., Pavlíková M., Žumár J., Černý R. (2014). Experimental Investigation of the Properties of Lime-Based Plaster-Containing PCM for Enhancing the Heat-Storage Capacity of Building Envelopes. Int. J. Thermophys..

[33] Mukhopadhyaya P., Kumaran K., Lackey J., Van Reenen D., Kumaran M., Dean S.W., Mukhopadhyaya P. (2007). Water Vapor Transmission Measurement and Significance of Corrections. J. ASTM Int..

[34] (2016). Hygrothermal Performance of Building Materials and Product Determination of Water Vapour Transmission Properties.

[35] Petersen P.E., Mukhopadhyaya P., Kumaran M., Lackey J. (2005). Use of the Modified Cup Method to Determine Temperature Dependency of Water Vapor Transmission Properties of Building Materials. J. Test. Eval..

[36] Jian F., Divagar D., Mhaiki J., Jayas D.S., Fields P.G., White N.D.G. (2018). Static and dynamic methods to determine adsorption isotherms of hemp seed with different percentages of dockage. Food Sci. Nutr..

[37] Ben Abdelhamid M., Mihoubi D., Sghaier J., Bellagi A. (2016). Water Sorption Isotherms and Thermodynamic Characteristics of Hardened Cement Paste and Mortar. Transp. Porous Media.

[38] (2002). Methods of Test for Mortar for Masonry-Part 18: Determination of Water Absorption Coefficient Due to Capillarity Action of Hardened Mortar.

[39] (2020). Natural Stone Test Methods-Determination of Resistance to Salt Crystallization.

[40] Lubelli B., van Hees R.P.J., Nijland T.G. Salt crystallization damage: How realistic are existing ageing tests? In Proceedings of the 1st International Conference on Ageing of Materials & Structures Delft University of Technology, Delft, The Netherlands, 26–28 May 2014.

[41] Granneman S.J., Lubelli B., Van Hees R.P. (2019). Effect of mixed in crystallization modifiers on the resistance of lime mortar against NaCl and Na_2_SO_4_ crystallization. Constr. Build. Mater..

[42] (2016). Specification for Mortar for Masonry-Part 1: Rendering and Plastering Mortar.

[43] WTA Merkblatt 2-9-04/D (2005). Sanierputzsysteme, Wissenschaftlich-Technische Arbeitsgemeinschaft für Bauwerkserhaltung und Denkmalpflege.

[44] Freire M.T., Veiga M.D.R., Silva A.S., de Brito J. (2019). Studies in ancient gypsum based plasters towards their repair: Physical and mechanical properties. Constr. Build. Mater..

[45] Romera J.I., Martínez-Ramírez S., Lapuente P., Blanco-Varela M.T., Mercadal M.P.L. (2013). Assessment of the physico-mechanical behaviour of gypsum-lime repair mortars as a function of curing time. Environ. Earth Sci..

[46] Fusade L., Viles H., Wood C., Burns C. (2019). The effect of wood ash on the properties and durability of lime mortar for repointing damp historic buildings. Constr. Build. Mater..

[47] Roels S., Carmeliet J., Hens H., Adan O., Brocken H., Cerny R., Pavlik Z., Hall C., Kumaran K., Pel L. (2004). Interlaboratory comparison of hygric properties of porous building materials. J. Therm. Envel. Build. Sci..

[48] Pavlíková M., Zemanová L., Záleská M., Pokorný J., Lojka M., Jankovský O., Pavlík Z. (2019). Ternary blended binder for pro-duction of a novel type of lightweight repair mortar. Materials.

[49] Pavlík Z., Pokorný J., Pavlíková M., Zemanová L., Záleská M., Vyšvařil M., Žižlavský M. (2019). Mortars with cushed lava granulate for repair of damp historical buildings. Materials.

[50] Quenard D., Sallee H. (1992). Water vapour adsorption and transfer in cement-based materials: A network simulation. Mater. Struct..

[51] Johannesson B.F. (2002). Prestudy on diffusion and transient condensation of water vapour in cement mortar. Cem. Concr. Res..

[52] Arizzi A., Cultrone G. (2014). The water transfer properties and drying shrinkage of aerial lime-based mortars: An assessment of their quality as repair rendering materials. Environ. Earth Sci..

[53] Chennouf N., Agoudjil B., Boudenne A., Benzarti K., Bouras F. (2018). Hygrothermal characterization of a new bio-based con-struction material: Concrete reinforced with date palm fibers. Constr. Build. Mater..

[54] Silva B., Pinto A.F., Gomes A. (2014). Influence of natural hydraulic lime content on the properties of aerial lime-based mortars. Constr. Build. Mater..

[55] Nenadálová S., Balík L., Rydval M., Bittner T. (2016). Laboratory Verification of Water Vapour Permeability of Plaster Compositions. Procedia Eng..

[56] Vares O., Ruus A., Raamets J., Tungel E. (2017). Determination of hygrothermal performance of clay-sand plaster: Influence of covering on sorption and water vapour permeability. Energy Procedia.

[57] Vares M.-L., Ruus A., Nutt N., Kubjas A., Raamets J. (2021). Determination of paper plaster hygrothermal performance: Influence of different types of paper on sorption and moisture buffering. J. Build. Eng..

[58] Mazhoud B., Collet F., Pretot S., Chamoin J. (2016). Hygric and thermal properties of hemp-lime plasters. Build. Environ..

[59] Thommes M., Kaneko K., Neimark A.V., Lovier J.P., Rodriguez-Reinoso F., Rouquerol J., Sing K.S.W. (2015). Physisorption of gases, with special reference to the evaluation of surface area and pore size distribution (IUPAC Technical Report). Pure Appl. Chem..

[60] Pavlík Z., Fořt J., Pavlíková M., Pokorný J., Trník A., Černý R. (2016). Modified lime-cement plasters with enhanced thermal and hygric storage capacity for moderation of interior climate. Energy Build..

[61] Pavlík Z., Žumár J., Medved I., Černý R. (2012). Water Vapor Adsorption in Porous Building Materials: Experimental Measurement and Theoretical Analysis. Transp. Porous Media.

[62] Barsoti E., Tan S.P., Piri M., Chen J.-H. (2020). Capillary-condensation hysteresis in naturally-occuring nonoporous media. Fuel.

[63] Extrand C. (1998). A Thermodynamic Model for Contact Angle Hysteresis. J. Colloid Interface Sci..

[64] Derluyn H., Derome D., Carmeliet J., Stora E., Barbarulo R. (2012). Hysteretic moisture behavior of concrete: Modelling and anal-ysis. Cem. Concr. Res..

[65] Bessadok A., Marais S., Roudesli S., Lixon C., Métayer M. (2008). Influence of chemical modifications on water-sorption and me-chanical properties of Agave fibres. Compos. Part A Appl. Sci. Manuf..

