# Supplementary material for: Lightweight Vapor-Permeable Plasters for Building Repair Detailed Experimental Analysis of the Functional Properties
Source: Materials (Basel). 2021 May 17;14(10):2613. doi: 10.3390/ma14102613 (PMC8156439; doi:10.3390/ma14102613)
Supplement: Supplementary file 1 [file materials-14-02613-s001.zip › materials-1212940-supplementary.pdf]

materials

MDPI

Supplementary Material

Lightweight Vapor-Permeable Plasters for Building Repair Detailed Experimental Analysis of the Functional Properties

Martina Záleská [1], Milena Pavlíková [1], Adam Pivák [1], Anna-Marie Lauermannová[2], Ondřej Jankovský[2] and Zbyšek Pavlík [1,*]

[1] Department of Materials Engineering and Chemistry, Faculty of Civil Engineering, Czech Technical University in Prague, Thákurova 7, 166 29 Prague 6, Czech Republic; martina.zaleska@fsv.cvut.cz (M.Z.); milena.pavlikova@fsv.cvut.cz (M.P.); adam.pivak@fsv.cvut.cz (A.P.)

[2] Department of Inorganic Chemistry, Faculty of Chemical Technology, University of Chemistry and Technology, Technická 5, 166 28 Prague 6, Czech Republic; anna-marie.lauermannova@vscht.cz (A.-M.L.); ondrej.jankovsky@vscht.cz (O.J.)

* Correspondence: pavlikz@fsv.cvut.cz; Tel.: +420-224-354-371

Citation: Záleská, M.; Pavlíková, M.; Pivák, A.; Lauermannová, A.-M.; Jankovský, O.; Pavlík, Z. Lightweight Vapor-Permeable Plasters for Building Repair Detailed Experimental Analysis of the Functional Properties. *Materials* **2021**, *14*, 2613. https://doi.org/10.3390/ma14102613

Academic Editor: Neven Ukrainczyk

Received: 22 April 2021
Accepted: 14 May 2021
Published: 17 May 2021

Publisher's Note: MDPI stays neutral with regard to jurisdictional claims in published maps and institutional affiliations.

Copyright: © 2021 by the authors. Submitted for possible open access publication under the terms and conditions of the Creative Commons Attribution (CC BY) license (http://creativecommons.org/licenses/by/4.0/).

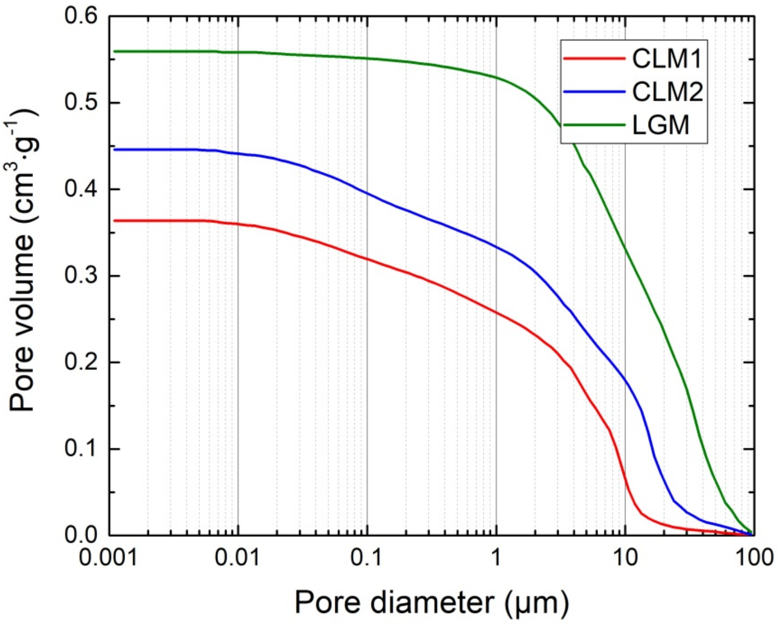

Figure S1. Cumulative pore volume distribution of the researched plasters.

Table S1. The pore size distribution parameters.

Pore Diameter Range (μm)	Relative Volume ($cm^3·g^{-1}$)			Relative Volume (%)			Porosity (%)		
	**CLM1**	**CLM2**	**LGM**	**CLM1**	**CLM2**	**LGM**	**CLM1**	**CLM2**	**LGM**
100–10	0.05342	0.17374	0.32328	14.67	38.87	57.31	7.136	21.004	34.251
10–1.0	0.25587	0.33153	0.52763	55.58	35.3	36.22	27.044	19.076	21.65
1.0–0.1	0.31811	0.39369	0.55093	17.09	13.91	4.13	8.314	7.514	2.469
0.1–0.001	0.35946	0.44068	0.55831	11.35	10.51	1.31	5.523	5.681	0.782
0.001–0.0010	0.3637	0.44579	0.55938	1.16	1.15	0.19	0.566	0.619	0.113

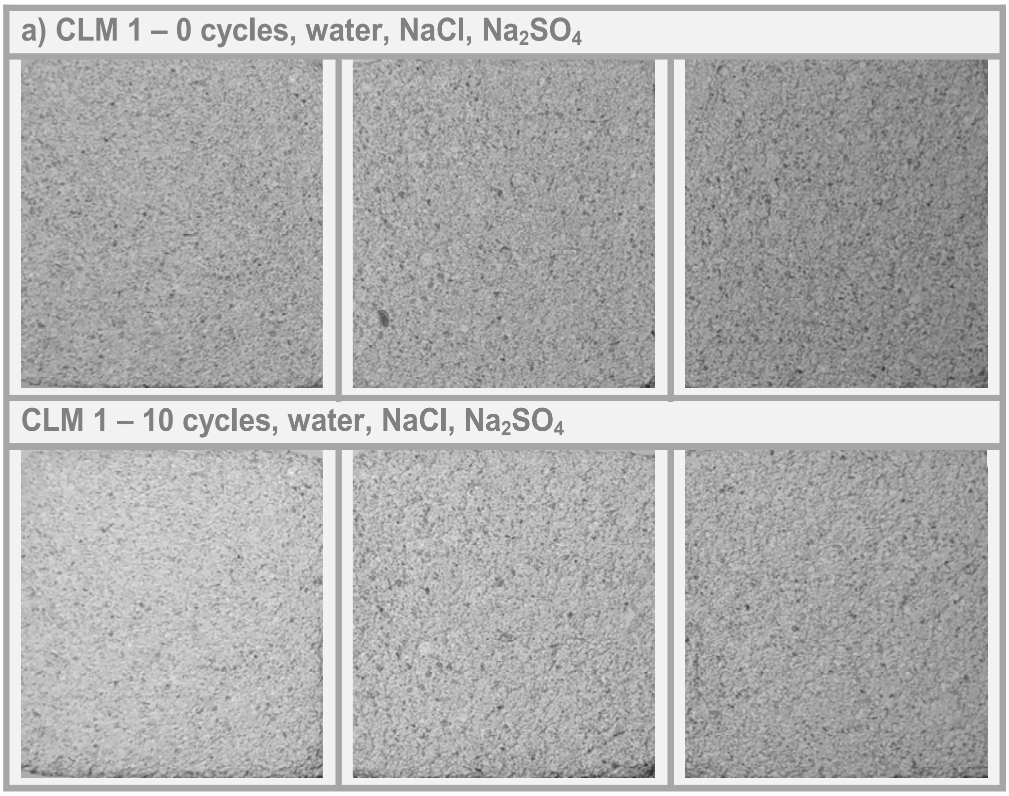

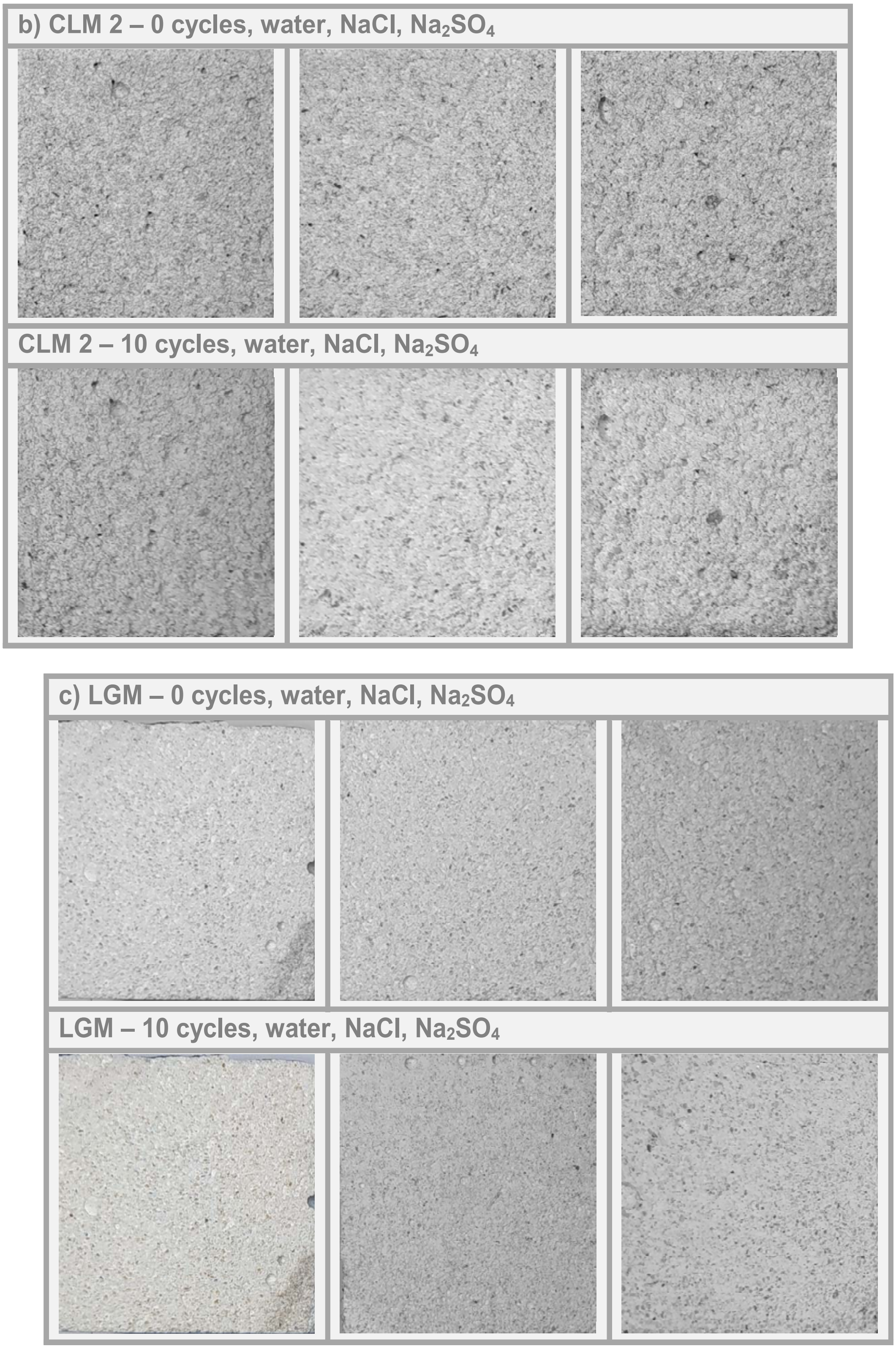

Figure S2. Photos of the researched plasters before and after salt crystallization test: (**a**) Plaster CLM1; (**b**) Plaster CLM2; (**c**) Plaster LGM.
